# Supplementary material for: Identification of High-Risk Groups in Urinalysis: Lessons from the Longitudinal Analysis of Annual Check-Ups
Source: Healthcare (Basel). 2022 Sep 6;10(9):1704. doi: 10.3390/healthcare10091704 (PMC9498401; doi:10.3390/healthcare10091704)
Supplement: Supplementary file 1 [file healthcare-10-01704-s001.zip › healthcare-1883547-supplementary.pdf]

Figure S1 Prevalence of proteinuria at fourth year as the number of the proteinuria at initial three years.

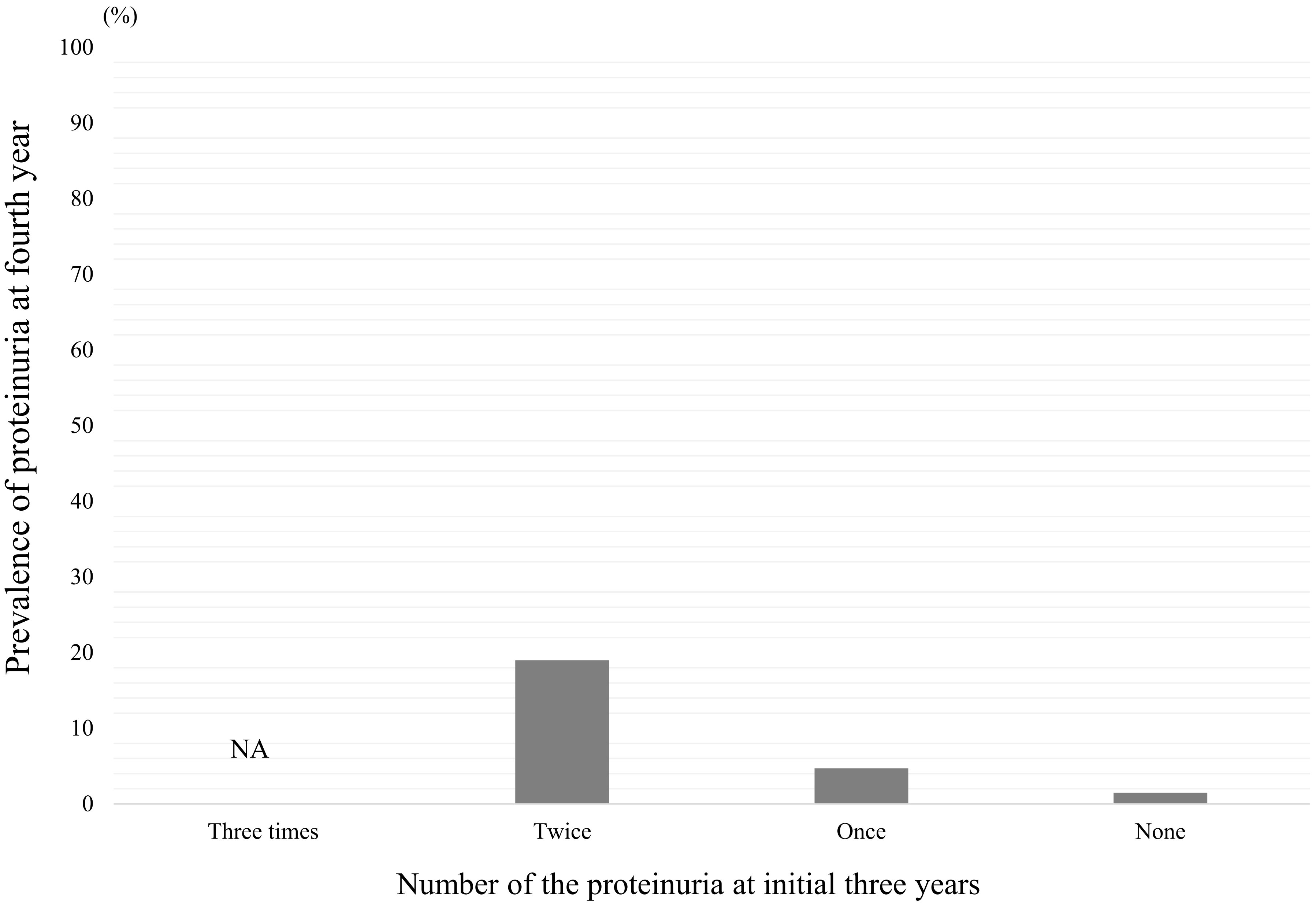

NA：Not Applicable

Figure S2 Prevalence of hematuria at fourth year as the number of the proteinuria at initial three years.

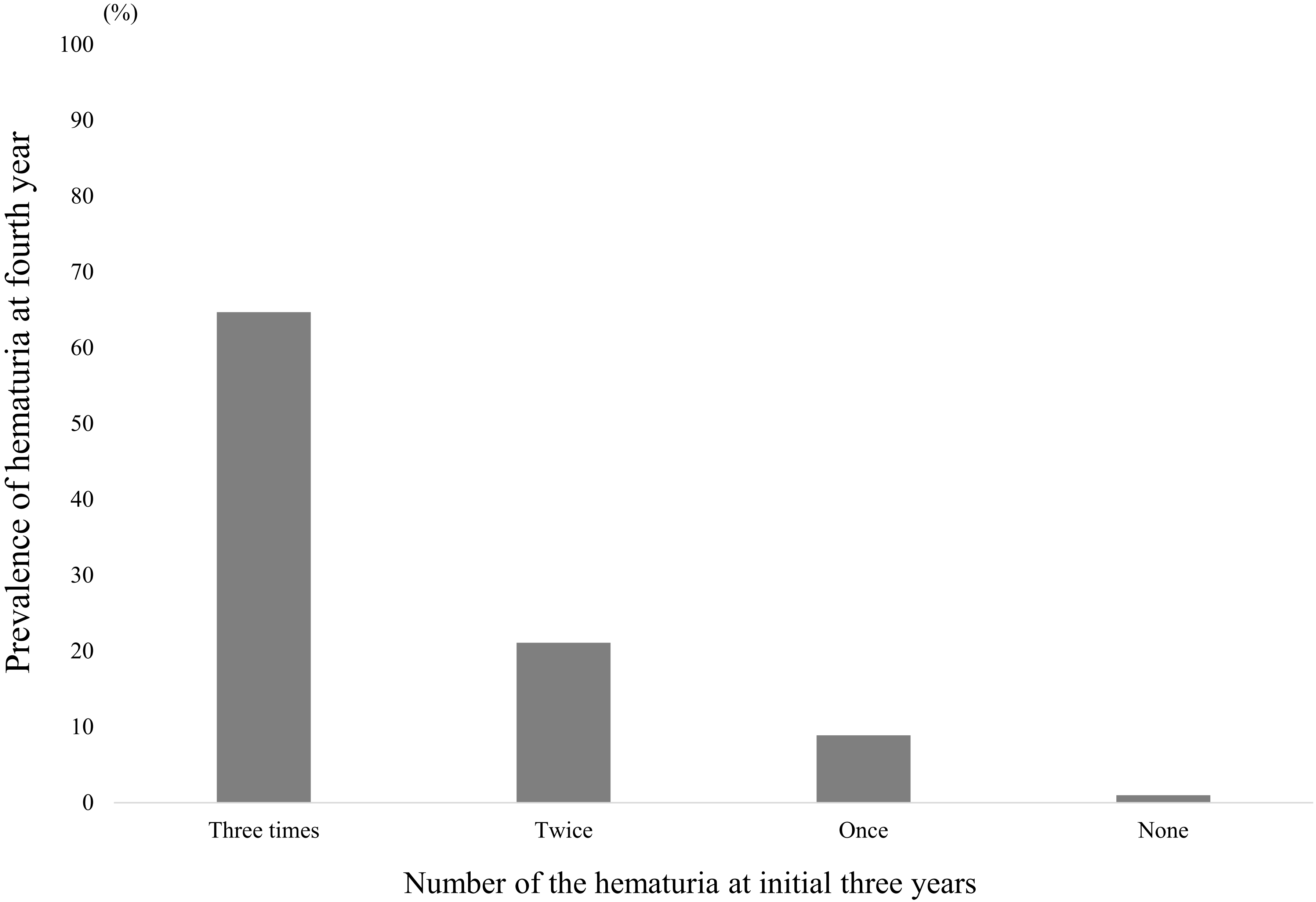

Figure S3 Prevalence of glucosuria at fourth year as the number of the proteinuria at initial three years.

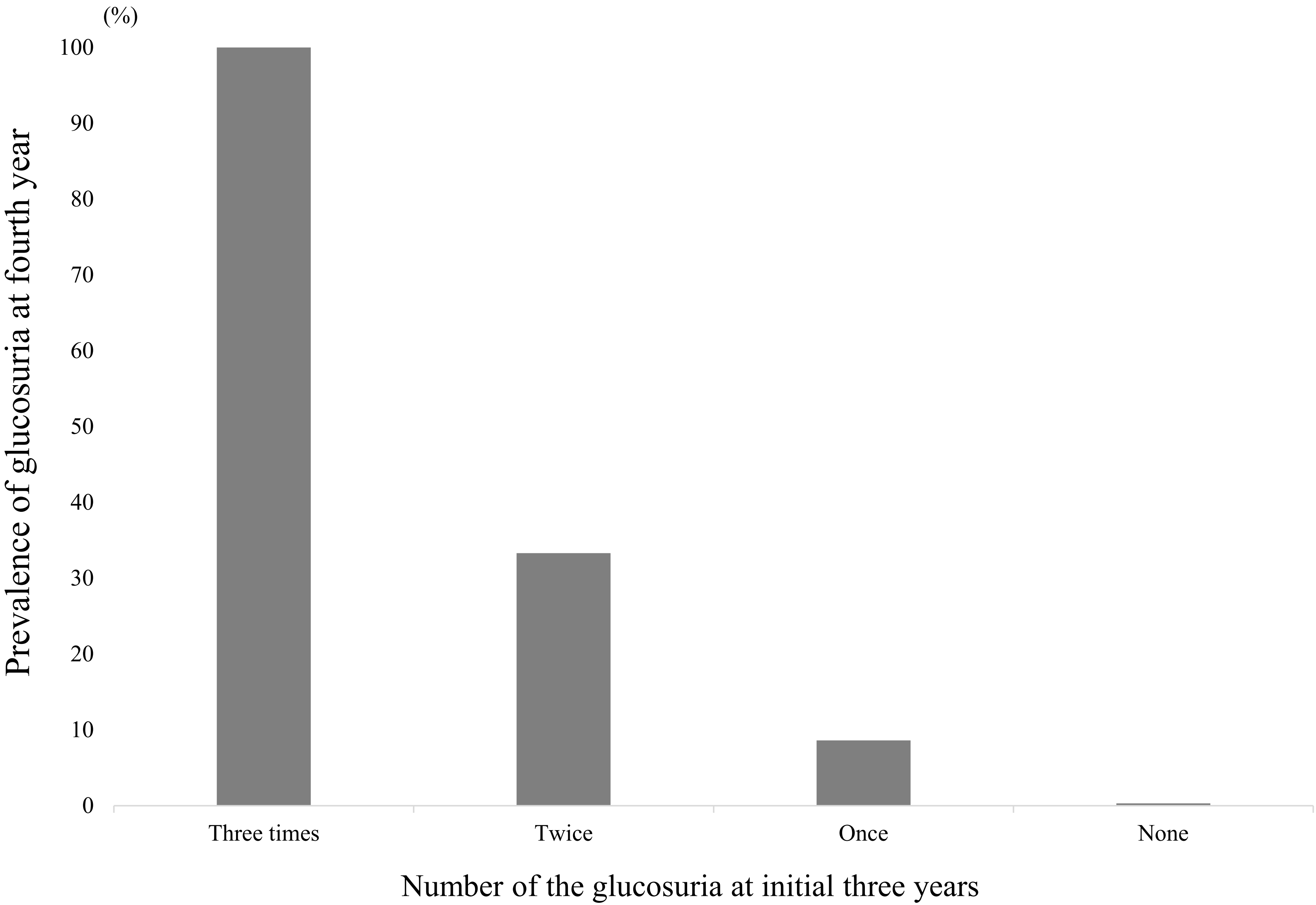

Supplementary Table: Number of the participants according to patterns of urinary abnormalities.

Patterns of urinary abnormalities (+: presence of urinary abnormality, −: absence of urinary abnormality)	Proteinuria All (n=13640) (n)	Proteinuria Men (n=10877) (n)	Proteinuria Women (n=2763) (n)	Haematuria All (n=13640) (n)	Haematuria Men (n=10877) (n)	Haematuria Women (n=2763) (n)	Glucosuria All (n=13640) (n)	Glucosuria Men (n=10877) (n)	Glucosuria Women (n=2763) (n)
+ + + +	0	0	0	11	7	4	4	4	0
+ + + −	4	3	1	6	5	1	0	0	0
+ + − +	2	1	1	4	2	2	2	2	0
+ + − −	8	6	2	23	13	10	5	5	0
+ − + +	0	0	0	4	3	1	2	1	1
+ − + −	3	2	1	13	4	9	6	5	1
− + + +	2	1	1	7	1	6	2	2	0
− + + −	6	5	1	20	5	15	1	0	1
+ − − +	4	3	1	4	0	4	1	1	0
+ − − −	179	146	33	119	27	92	26	22	4
− + − +	10	9	1	18	5	13	6	5	1
− + − −	228	185	43	136	31	105	48	45	3
− − + +	14	11	3	15	3	12	5	5	0
− − + −	163	132	31	124	28	96	54	49	5
− − − +	193	158	35	135	23	112	43	36	7
− − − −	12824	10215	2609	13001	10720	2281	13435	10695	2740
